# Glutamate Signaling Pathway in Absence Epilepsy: Possible Role of Ionotropic AMPA Glutamate Receptor Type 1 Subunit

**DOI:** 10.22037/ijpr.2020.112638.13869

**Published:** 2020

**Authors:** Fahime Zavvari, Sayed Mostafa Modarres Mousavi, Maryam Ejlali, Shahram Barfi, Fariba Karimzadeh

**Affiliations:** a *Cellular and Molecular Research Center, Iran University of Medical Sciences, Tehran, Iran. *; b *Shefa Neuroscience Research Center, Khatam Alanbia Hospital, Tehran, Iran. *; c *Department of Nano biotechnology, Faculty of Biological Sciences, Tarbiat Modares University, Tehran, Iran. *; d *Department of Virology, School of Public Health, International Campus, Tehran University of Medical Sciences, Tehran, Iran.*

**Keywords:** Epilepsy, Brain, Somatosensory cortex, Seizure, Glutamate

## Abstract

AMPA receptors, consisting of glutamate receptor type1 (GluR1) subunit are involved in the pathophysiology of some neurological disorders. In this study, the role of the GluR1 subunit in the development, as well as features of absence seizures were assessed. Both Wistar and WAG/Rij (a genetic animal model of absence epilepsy) rats with 2 and 6-month ages were included in the study. The expression of GluR1 was measured in the somatosensory cortex. Moreover, the effects of pharmacological activation and inhibition of AMPA receptors on the characteristic of absence epileptic activities were evaluated by microinjection of agonist or antagonist of AMPA receptors on the somatosensory cortex in the epileptic WAG/Rij rats. Distribution of the GluR1 subunit of AMPA receptors in the both IV (*p < *0.001) and VI (*p < *0.01) layers of the somatosensory cortex in the epileptic WAG/Rij rats was higher than non-epileptic animals. In addition, the microinjection of AMPA receptors agonist on the somatosensory cortex of the WAG/Rij rats increased both amplitude (*p < *0.01) and duration (*p < *0.001) of spike-wave discharges (SWDs), while injection of antagonist reduced amplitude (*p < *0.001) and duration (*p < *0.01) of SWDs in the somatosensory cortex of epileptic rats. The high expression of GluR1 in the somatosensory cortex of epileptic rats suggests the role of AMPA receptors consisting of the GluR1 subunit in the development of absence seizures. The modulatory effects AMPA receptors on the feature of SWDs suggest the potential of AMPA receptors antagonists as a therapeutic target for absence epilepsy.

## Introduction

Absence epilepsy is considered as non-convulsive epilepsy that is characterized by a sudden loss of consciousness and is associated with generalized spontaneous and synchronous bilateral spike-wave discharges (SWDs) in the electroencephalogram (1). 

It has been reported that the intra-thalamic neuronal networks originate spike-waves discharges in the absence epilepsy (2, 3). The role of the somatosensory neocortex to trigger and generation of SWDs has been reported (4-7).

The triggering role of the somatosensory cortex has been identified in the SWDs initiation in the Wistar Albino Glaxo from Rijswijk (WAG/Rij) rats (8). Analysis of cortical and thalamic electroencephalographic recordings from WAG/Rij rat, a valid and naturally occurring genetic model of absence epilepsy revealed that SWDs were initiated in the primary somatosensory cortex and then rapidly propagated to motor cortices and thalamic nuclei (9). 

The somatosensory cortex is typically organized in six layers, beginning with layer I nearby the brain surface and extending progressively deeper to layer VI. The incoming sensory signals first enter the neuronal layer IV, and then propagate to the surface of the cortex and deeper layers. The processed signals are sent to the thalamus from layer VI. These signals help to adjust the excitation of thalamic nuclei (10, 11). 

The ictogenic neurons in deep layers of the somatosensory cortex display fast activation, hyperexcitability and hyper synchronization in the absence seizures (4, 5). 

The α-amino-3-hydroxy-5-methyl-4-isoxazole propionic acid receptors (AMPARs) modulate cortical excitability (12). These receptors are a subtype of the ionotropic glutamate receptor with intrinsic cation permeable channels and mediate fast synaptic transmission in the central nervous system (CNS). AMPARs are abundant and distributed widely in the CNS, although there are regional density differences (13). Four subtypes were designated as GluR1, GluR2, GluR3, and GluR4 for AMPARs (14). The GluR1 and GluR3 subunits show a significant Ca^2+^ conductance, whereas the GluR2 subunit is impermeable to Ca^2+ ^(15). 

The role of AMPARs have been determined in the pathogenesis of many neurological and neurodegenerative disorders, such as biological manifestations of dementia in Alzheimer’s disease or neuronal death in the amyotrophic lateral sclerosis and stroke (16).

Furthermore, AMPARs play a key role in the synchronization, generation and spread of epileptic discharges in the cortex, amygdala, and thalamus (17, 18).

Since the GluR1 subunit of AMPA receptors shows a significant Ca^2+^ conductance, and given the fact that the main evoking event in absence epilepsy is rise of intracellular calcium ions in brain regions related to epilepsy, in this study we assessed the alternation of GluR1 expression in the deep layers of somatosensory cortex during development of absence seizures as well as modulatory effect of AMPARs on the SWDs features. 

## Experimental

*Animals*

Male WAG/Rij and Wistar rats were housed under conditions with temperature 22 ± 2 °C, 12-h light/dark cycle and free access to food and water. All experiments were performed in accordance with the guidelines approved by the animal ethics of Shefa Neuroscience Research Center, Tehran, Iran. The animals were divided into four groups of both strains with 2 and 6 months of age (n = 6). 

*Electrocorticoencephalogram (ECoG) Recordings*

To select epileptic animals, ECoG was recorded at least for 6 hours. All the animals were anesthetized by intraperitoneal chloral hydrate injection (350 mg/kg; Sigma–Aldrich, Munich, Germany) and recording electrodes were implanted stereotaxically on the dura mater of parietal cortices. Silver electrodes connected to an amplifier (EXT-02 F; NPI, Germany) and ECoG were stored by a digital oscilloscope. Sedation was retained by repeated injections of fentanyl every 20-30 min (0.033 mg/kg per hour, i.p.; (11, 19)). At the age of 6 months, WAG/Rij rats exhibited most SWDs discharges and were included in the study as epileptic rats (9). Most of the two-month-old WAG/Rij rats (before adulthood) did not display SWDs and have been considered as a pre-symptomatic group. 

*Immunofluorescent staining*

After deep anesthesia with 350 mg/kg chloral hydrate (Sigma–Aldrich) and transcardial perfusion of 200 mL of saline and then 400 mL of 4% paraformaldehyde solution (PFA), the brains were removed and inserted in the 4% PFA for 1 week at 4 °C.

Being followed by preparing paraffin-embedded blocks, the serial coronal sections in AP = 1.8–3.3 mm posterior to the bregma (Paxinos and Watson, 1998) with 8μm thickness were cut by microtome (20). Five slices from each animal were selected for immunofluorescent staining. 

The sections were rehydrated through series of xylol and alcohol and washed with phosphate-buffered saline (PBS; pH 7.4) three times and incubated in blockage solution (3% H2O2/ methanol for 5 min). The sections were boiled in citrate buffer (pH 6.0) at 95 °C for 10 min and were cooled at room temperature. The sections were incubated in 10% normal goat serum and 0.2% Triton-X100 for 1 h. The sections were incubated overnight at 4 °C with mouse monoclonal anti-rat antibody (sc-55509, Santa Cruz) against GluR1 overnight, diluted at 1:350 in a solution comprising 1–5% NGS in 0.3% Triton X-100 and 0.1 M PBS at pH of 7.4. The sections were rinsed in PBS three times and were incubated with FITC goat anti-mouse secondary antibody (sc-2010, Santa Cruz). Secondary antibody concentration was 1:100 in PBS with 0.3% Triton X-100 and 5% NGS at 22 °C for 1 h. After several times washing with PBS, nuclei were counterstained by 5 min at 37 °C submerging sections in a propidium iodide solution (75 μg/mL). The sections were mounted on a coverslip with glycerol 90% mounting buffer and were observed with a fluorescent microscope (Olympus, Japan). Control for the specificity of immunostaining was performed by the omission of the primary antibody and its substitution for normal mouse serum. Images (five visual field / section) for analysis were taken with a digital camera attached to the fluorescent microscope (40× objective). The number of cells reacted with the GluR1 antibody was counted per 1 mm^2^ in the IV and VI layers of the somatosensory cortex by using ImageJ software. 

*Drugs Application Procedures*

Experiments were performed on adult 6-month-old WAG/Rij (epileptic) rats. The animals were divided into three groups: vehicle, agonist, and antagonist (n = 6). The procedures of electrode implementation and ECoG recording have been mentioned above. A hole was created on the skull bone (AP = -2.5 mm, ML = ±4.3 mm) according to the coordinates of the rat brain atlas by Paxinos and Watson (1998) for local drug infusion on the somatosensory cortex.

ECoG was recorded for 30 min as a baseline activity. Selective AMPA agonist (Rs-AMPA hydrobromide; Tocris, Cat. No. 1074) was injected in the agonist group. Selective non-NMDA (N-Methyl-D-aspartate) ionotropic glutamate receptors antagonist (DNQX, Tocris, Cat. No. 2312) was injected in the antagonist group. Distilled water was injected in the vehicle group. The injections were performed by using a 10-gaged Hamilton syringe on the primary somatosensory cortex (21). The drugs were dissolved in distilled water and microinjected in a concentration of 0.5 μg/0.5 μL for 2 min (22, 23). 

ECoG monitoring was continued for 2 h followed by drug infusion. By using the AxoScope 10 software (Axon Instruments, USA) the amplitude, frequency, and duration of SWDs were analyzed. 

*Statistical Analysis*

Data were expressed as Mean ± SEM. The immunohistochemical statistical analysis was carried out by one-way analysis of variance (ANOVA) followed by Tukey’s *post hoc* test. The frequency, amplitude, and duration of SWDs were statistically analyzed before and after usage of drugs by Friedman and Wilcoxon test. The significant point was considered *p < *0.05. The PASW Statistics 18 was applied for statistical analysis.

## Results

*Cortical distribution of GluR1*

**Figure 1:**
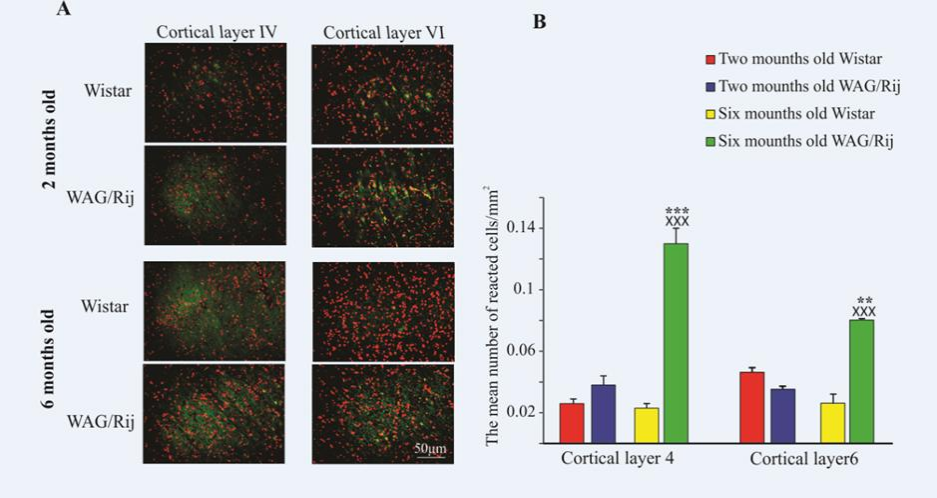
Distribution of GluR1 in the somatosensory cortex of two- and six-month-old WAG/Rij and Wistar rats. (A) Photomicrographs of GluR1 distribution in the somatosensory cortex of two- and six-month-old WAG/Rij and Wistar rats. (B) The bar graph shows quantitative results of GluR1 distribution in the somatosensory cortex. GluR1 highly expressed in the both layers of somatosensory cortex in the 6-old-month WAG/Rij rats. ^×××^ indicates *p < *0.001 compared to Wistar rats. ^***^ and ^**^ indicate *p < *0.01 and *p < *0.001 respectively compared to 2-old-month WAG/Rij rats

The data are presented as the mean number of reacted neurons/mm^2 ^(Figure 1A). GluR1 highly was expressed in the 6-month-old WAG/Rij rats compared to 2-month-old rats of the same race both in IV layer (0.13 ± 0.01) and in VI layers (0.08 ± 0.001) of the somatosensory cortex (*p < *0.001 and *p < *0.01, respectively, Figure 1B). Furthermore, the distribution of GluR1 in the 6-month-old WAG/Rij was more than 6- and 2-month-old Wistar rats in both layers of cortex (*p < *0.001, Figure 1B). The mean number of reacted neurons was 0.026 ± 0.003 in layer IV and 0.046 ± 0.003 in layer VI of the 2-month-old Wistar rats. The mean number of reacted neurons was 0.023 ± 0.003 in layer IV and 0.026 ± 0.006 in the layer VI of 6-month-old Wistar rats.

There was no significant difference between 6- and 2-month- old Wistar rats in both layers of the somatosensory cortex (Figure 1B, *p* = 0.9).

*AMPARs modulatory effects on the absence seizures*

**Figure 2:**
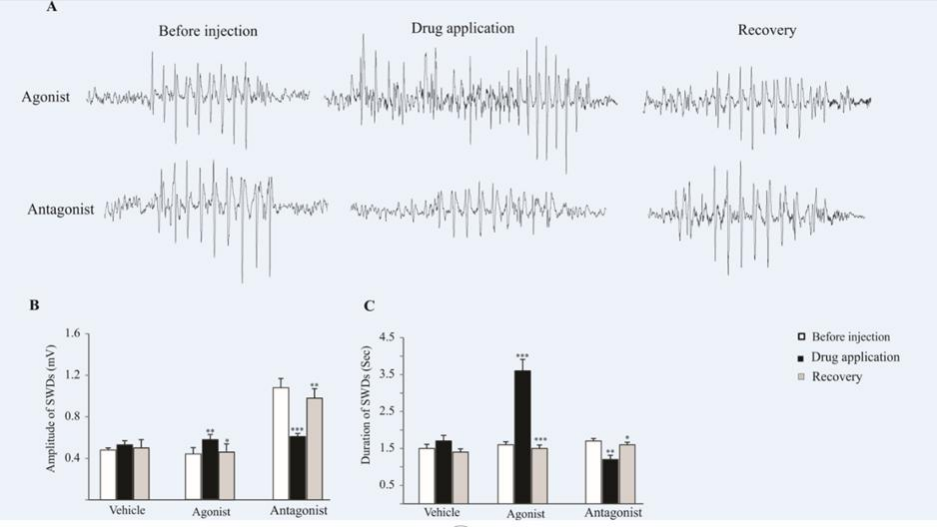
The effect of AMPARs agonist and antagonist on the SWDs. (A) ECoG traces were recorded before, during and after drug administration. (B and C) Bar charts summarized mean ± SEM of SWDs amplitudes and durations. Micro injection of agonist significantly increased the duration (*p < *0.001) and amplitude (*p < *0.01) of SWDs in comparison with before injection. Administration of AMPARs antagonist on the somatosensory cortex reduced the amplitude (*p < *0.001) and duration (*p < *0.01) of SWDs in comparison with before injection. In 2 h after injection (recovery time) the amplitude and duration of SWDs in agonist (*p < *0.05 for amplitude and *p < *0.001 for duration) and antagonist (*p < *0.01 for amplitude and *p < *0.05 for duration) groups has changed significantly compared to injection time and reached near the initial values

Six-month-old WAG/Rij rats were monitored under neuroleptic anesthesia during the recording of ECoG. The bilaterally synchronized SWDs at 5–9 Hz on the ECoG were started and ended abruptly on a normal background pattern. The agonist and antagonist of AMPA receptors were microinjected unilaterally on the somatosensory cortex (Figure 2). 

As we have shown in Figure 2B, the amplitude of SWDs increased (0.57 ± 0.05 mV) significantly after the microinjection of AMPARs agonist (*p < *0.01), while, the amplitude of SWDs decreased significantly after the microinjection of the AMPARs antagonist (*p < *0.001). The mean amplitude of SWDs was 0.61 ± 0.03 mV before the injection of antagonist and 1.2 ± 0.11 mV after injection. In 2 hours after injection (recovery time) the amplitude of SWDs in agonist (*p < *0.05) and antagonist (*p < *0.01) groups has changed significantly compared to injection time (Figure 2B). 

In addition, the duration of SWDs significantly increased (3.6 ± 0.31 s) after the application of AMPARs agonist (*p < *0.001) while, following the antagonist infusion, the duration of SWDs significantly decreased (1.2 ± 0.11 S, *p < *0.01) compared to before and after (recovery time) drug administration (Figure 2C). In recovery time the duration of SWDs in agonist (*p < *0.001) and antagonist (*p < *0.05) groups has changed significantly compared to injection time (Figure 2B). 

There was no significant difference in the frequency of SWDs following the agonist and antagonist infusion.

## Discussion

*Cortical dis excitation developed absence seizures*

Developmental alternations in the cortical excitation and/or inhibition are involved in the pathophysiology of some neurodevelopmental disorders (24, 25). Cortical disinhibition has been indicated in the pathogenesis of Parkinson’s disease (26). Cortical enhancement of excitability has a key role in the pathophysiology mechanism of Alzheimer’s disease. Further, cortical excitability might lead to cortical dysfunction in the primary lateral sclerosis as well as amyotrophic lateral sclerosis (27, 28). It has been reported that the fluctuation of cortical excitation underlies hemiplegic attacks (29). High resting, active motor threshold and small evoked potentials in patients with Sydenham’s chorea emphasized the low excitability of corticospinal output (30). 

Epilepsy as a neurodevelopment disorder has been investigated for cortical anomalies.

We evaluated the changes of the GluR1 subunit in the somatosensory cortex during the development of absence epilepsy. 

WAG/Rij rats display SWDs in adulthood and are considered as a valid genetic model of gene-linked absence epilepsy (9). Our findings indicated that the GluR1 subunit in the somatosensory cortex, in both IV and VI cortical layers, has been highly expressed in the adult epileptic rats. On the contrary, it has been down-regulated in the adult healthy Wistar rats.

GluR1 as a Ca^2+ ^permeable subunit of AMPARs is important for normal synaptic function after birth. In early developmental stages, many synapses contain GluR1, start to change into GluR2 (Ca^2+ ^impermeable subunit of AMPARs) during the age-dependent development of cortical layers (31, 32). 

Abnormalities of structure and function of synapses during the development of CNS are involved in the pathogenesis of absence seizures (25). Prior studies have reported high expression of AMPA receptors in the hippocampus and cerebral cortex in the epileptic people and rats (33-35). In the hippocampus of epileptic people, the density of AMPA GluR1 subunit proteins has been increased (36). GluR2 markedly has been down-regulated in the neurons that were subject to epileptic seizures (37). Further investigations documented that enhancement of hippocampal GluR1 followed by some neuropathological conditions such as inflammation (38) or impaired autophagy, reduced GluR1 degradation and increased seizure susceptibility in the adult rats (39, 40). Functional and single-cell transcript analyses have shown that prolonged receptor responses in the hippocampal astrocytes of epileptic patients, resulted from high expression of GluR1 in this region (41-43). 

These data suggest that aberrant patterns of physiological activities influence the composition of AMPA receptor subunits in a region-specific and/or cell-type-specific manner. 

Synaptic activity is needed for synaptic delivery AMPA receptors containing the GluR1 subunit, whereas GluR2 is constitutively inserted into synapses (44). It is previously was shown that the expression of the stargazin, a critical regulators of the trafficking and function of AMPA receptors, increased in the somatosensory cortex of genetic absence epilepsy rat from Strasbourg (GAERS) (45); and its elevation is accompanied with an increase in AMPA receptor proteins (GluR1 and GluR2) in the somatosensory cortex of adult epileptic GAERS (46). It seems that AMPA receptor subunits in different neuronal populations are subject to distinct regulatory processes in response to intense physiological activity. 

Ictogenic neurons of deep layers of somatosensory cortex display distinctive hyperactivity that leads to these data suggesting that aberrant patterns of physiological activities influence the composition of AMPA receptor subunits in a region-specific and/or cell-type-specific manner. 

Synaptic activity is needed for synaptic delivery AMPA receptors containing the GluR1 subunit, whereas GluR2 is constitutively inserted into synapses (44). It is previously was shown that the expression of the stargazin, a critical regulator of the trafficking and function of AMPA receptors, increased in the somatosensory cortex of genetic absence epilepsy rat from Strasbourg (GAERS) (45); and its elevation is accompanied with an increase in AMPA receptor proteins (GluR1 and GluR2) in the somatosensory cortex of adult epileptic GAERS (46). It seems that AMPA receptor subunits in different neuronal populations are subject to distinct regulatory processes in response to intense physiological activity. 

Ictogenic neurons of deep layers of somatosensory cortex display distinctive hyperactivity that leads to the firing of distant cortical and thalamic cells during the epileptic discharges (5, 47 and 48). Based on these data we conclude that aberrant cortical expression of GluR1 receptors in epileptic rats may contribute to the development of SWDs.

*AMPARs modulatory effects on the absence seizures*

We assessed the effect of pharmacological modulation of AMPARs on the characteristic of bioelectrical brain activities in the somatosensory cortex of epileptic rats and showed that cortical activation of AMPARs exacerbated the absence seizures in the epileptic WAG/Rij rats.

Various pathophysiological mechanisms are involved in the abnormal neuronal discharges in epilepsy. Regardless of the primary cause, abnormal glutamate (excitatory) neurotransmission, affecting on metabotropic and ionotropic receptors, is was thought to be critical for seizure generation and the epileptic state (21, 49 and 50). 

Throughout the CNS, fast synaptic excitation within and between brain regions related to epilepsy is mediated mostly by AMPA and NMDA receptor localized to the postsynaptic membrane. It has been shown that activation of AMPA receptors can evoke seizures in the preclinical models (51). Based on previous studies, selective blockade of NMDA receptor alone is not sufficient to eliminate epileptiform discharges and AMPA receptors play a key role in modulating excitatory synaptic transmission in epilepsy (52, 53).

In addition, we showed that inactivation of AMPARs in the somatosensory cortex suppressed seizure activities in the absence epileptic rats.

The anti-epileptic effect of AMPA receptor antagonists has been reported. These components noticeably reduce or suppress epileptiform activity by inhibiting glutamate-mediated excitation. It was shown in *in-vitro* (54-57) as well as *in*-*vivo* animal models of seizure that AMPA receptor antagonists markedly reduce or abolish epileptiform activity (17, 58).

Our findings clarify the potential therapeutic role of AMPARs for absence seizures and in the modulation of cortical neural hyperactivity.
